# Comparative Outcomes of GH Treatment in Pediatric Idiopathic Short Stature and GH Deficiency

**DOI:** 10.1210/jendso/bvaf133

**Published:** 2025-08-16

**Authors:** Moshe Phillip, M Jennifer Abuzzahab, Alberto Pietropoli, Jean-Marc Ferran, Michael Højby, Nicky Kelepouris, Primož Kotnik, Michel Polak, Lars Sävendahl

**Affiliations:** Institute for Endocrinology and Diabetes, National Center for Childhood Diabetes, Schneider Children's Medical Center of Israel, Petah Tikva 4920235, Israel; Faculty of Medical and Health Sciences, Tel Aviv University, Tel Aviv 6997801, Israel; Diabetes and Endocrine Center, Children's Minnesota, Saint Paul, MN 55102, USA; Global Medical Affairs, Novo Nordisk Health Care AG Zurich 8058, Switzerland; Data Science, Qualiance ApS, Copenhagen 1663, Denmark; Clinical Drug Development, Novo Nordisk A/S, Søborg 2860, Denmark; US Medical Affairs, Rare Endocrine Disorders, Novo Nordisk Inc, Plainsboro, NJ 08536, USA; Department of Pediatrics, Medical Faculty, University of Ljubljana, Ljubljana 1000, Slovenia; Department of Pediatric Endocrinology, Diabetes and Metabolism, University Children's Hospital Ljubljana, University Medical Center, Ljubljana 1000, Slovenia; Pediatric Endocrinology, Gynecology and Diabetology, Hôpital Universitaire Necker Enfants Malades, Assistance Publique-Hôpitaux de Paris Université Paris Cité, INSERM U1016, Institut IMAGINE, Centre de Référence des Maladies Endocriniennes Rares de la Croissance et du Développement, Paris 75015, France; Pediatric Endocrinology, Karolinska University Hospital and Department of Women's and Children's Health, Karolinska Institutet Solna 171 77, Sweden

**Keywords:** idiopathic short stature, growth hormone deficiency, growth hormone response, growth hormone safety, somatropin

## Abstract

**Context:**

GH treatment in children with idiopathic short stature (ISS) can be controversial, and analyses comparing responses to children with GH deficiency (GHD) are limited.

**Objective:**

To compare the effectiveness and safety of GH treatment in children with ISS and GHD, including those reaching near adult height (NAH).

**Methods:**

This post hoc analysis of the NordiNet International Outcome Study (2006-2016) and the American Norditropin Studies: Web-Enabled Research Program (2002-2016) included children with ISS or GHD who initiated treatment aged <18 years. The safety analysis set had birthdate and GH exposure information. The effectiveness analysis set was GH-naïve with valid baseline information. GH exposure, effectiveness, and safety outcomes were analyzed annually for ≤10 years.

**Results:**

The safety analysis set included 3816 children with ISS and 22 858 with GHD. The effectiveness analysis set comprised 18 405 children (ISS: 2684; GHD: 15 721), 1856 of whom reached NAH (ISS: 230; GHD: 1626). Average dose of GH was higher for children with ISS vs children with GHD but mean duration of treatment was shorter. At NAH, height SD score (mean [SD]) was −1.21 (1.09) and −0.90 (1.20) for children with ISS and GHD, respectively, whereas change in height SD score (mean [SD]) from baseline to 10 years was 1.21 (0.86) and 1.45 (1.09). Incidence of adverse reactions was similar across indications, with no new safety signals.

**Conclusion:**

GH treatment over 5 to 10 years effectively increased height in children with ISS and children with GHD, including those who reached NAH, with a favorable benefit-risk profile.

Idiopathic short stature (ISS) is defined as short stature of unknown cause [1, 2]. A diagnosis of ISS is given when height is >2 SD scores (SDS) below the mean for a given age, sex, and population, after excluding underlying medical conditions or genetic differences that could contribute to short stature or slower growth [1, 3-5]. ISS accounts for a heterogeneous group of children who were normal size for gestational age at birth and have no evidence of systemic, endocrine, nutritional, or chromosomal abnormalities [4, 5]. Specifically, these children do not have a deficiency of GH [2].

Evidence suggests that children with ISS can achieve close to average adult height following GH treatment [6-8]. According to US Food and Drug Administration guidelines, US children must have a height SDS (HSDS) ≥ 2.25 below the mean for treatment consideration [9]. A meta-analysis of results from 21 studies of children with ISS indicated that GH treatment can improve short-term linear growth and increase adult height compared with no GH treatment [7]. Furthermore, a systematic review of 14 randomized trials concluded that GH treatment in children with ISS can reduce the deficit in height as adults [8]. Although there is limited evidence from clinical trials assessing whether height-promoting interventions in ISS can potentially reduce the psychosocial burden [10], observations in other trials report positive changes in different measures related to quality of life [10-14]. Despite that, use of GH in children with ISS remains controversial because diagnosis is descriptive, and treatment response is variable because of the heterogeneity of underlying causes [15-17]. Furthermore, in some cases identified initially as ISS, a partial resistance to GH may be present because of acquired or genetic causes [11]. Use of GH in children with ISS requires accurate determination of the underlying abnormality in the GH/IGF axis and the predicted adult height and bone age of the patient [16].

Of GH-treated children, most data focus on GH deficiency (GHD), and the consensus is that GH treatment is effective in aiding children with GHD to achieve both short-term growth and adult height [18-20]. Nevertheless, there is disagreement as to whether patients with GHD respond equally or more favorably to GH than patients with ISS [21-23]. In particular, few studies have compared effectiveness and safety of long-term GH treatment in children diagnosed with GHD and ISS [24]. Although Norditropin is approved for treatment of children with GHD in Europe, the United States, and Japan, it is only approved to treat ISS in the United States and South Korea [25-28].

The aim of this analysis was to compare effectiveness and safety of treatment with Norditropin (somatropin), a daily recombinant GH, in children with ISS and GHD, including those who reached near adult height (NAH).

## Materials and Methods

### Study Design

This post hoc analysis used data from 2 complementary, noninterventional, observational studies, NordiNet International Outcome Study (NordiNet IOS; NCT00960128; 2006-2016) and the American Norditropin Studies: Web-Enabled Research (ANSWER) Program (NCT01009905; 2002-2016). The design and methodology of both studies have been previously reported [29, 30].

NordiNet IOS and the ANSWER Program were approved by the relevant ethics committees and conducted with informed consent from parents or legal guardians of pediatric patients. Pseudonymization of data was performed in accordance with the Declaration of Helsinki, regulatory requirements, and Guidelines for Good Pharmacoepidemiology Practices.

### Study Population

**Figure 1.**
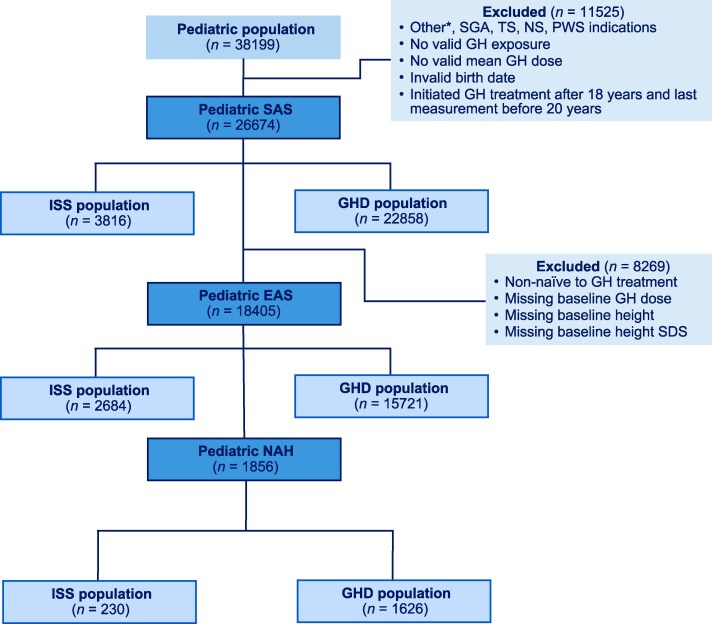
Disposition of patients with ISS and GHD included in the analyses. Abbreviations: CRD, chronic renal disease; EAS, effectiveness analysis set; GHD, GH deficiency; ISS, idiopathic short stature; NAH, near adult height; NS, Noonan syndrome; PWS, Prader-Willi syndrome; SAS, safety analysis set; SDS, SD score; SGA, small for gestational age; TS, Turner syndrome. *Pediatric patients with a diagnosis other than GHD, SGA, TS, CRD, ISS, NS, and PWS.

Pediatric patients enrolled in NordiNet IOS or ANSWER, treated with Norditropin and diagnosed with ISS or GHD, as reported by their physician, were included in this analysis (Fig. 1). The safety analysis set (SAS) included patients with birthdate information and previous GH exposure, regardless of GH brand. Children in the SAS initiated Norditropin before 18 years of age and were treated until epiphyseal closure. Children who initiated any GH treatment before 18 years of age and Norditropin after the age of 18 years were also included, provided that Norditropin treatment did not continue after 20 years of age or after epiphyseal closure.

Effectiveness was analyzed in children in the SAS who had valid baseline height, age, and dosing information and were treatment-naïve at the baseline visit (effectiveness analysis set [EAS]). A further analysis set included children in the EAS who achieved NAH, defined by height velocity <2 cm/year during the last year and chronological age >15 years for females, >16 years for males, or when chronological age was >18 years.

### Study Outcomes

The following effectiveness variables were calculated at baseline and annually, for up to 10 years of GH treatment in the EAS: GH exposure (mg/kg/day), height velocity SDS (HVSDS), HSDS, change in HSDS (ΔHSDS), IGF-I SDS, and bone age/chronological age ratio (BA/CA). IGF-I measurements were derived from local assays. Safety data were based on physicians’ reporting of adverse events. Adverse reactions (ARs; adverse events deemed related to the product) were subdivided into serious adverse reactions (SARs) and nonserious adverse reactions (NSARs).

### Statistical Analyses

Effectiveness outcomes were summarized using descriptive analyses (mean, SD, and p10/p90). Safety data were presented as incidence rates (IR) per 1000 patient-years of exposure (PYE) and as incidence rate ratios (IRR). Primary analyses included children grouped into ISS or GHD subgroups based on diagnosis reported by their physician.

Considering the large sample size included in this analysis and the difference in sample size between the 2 groups, *t*-tests were conducted to assess the statistical differences in means, and Cohen's d statistic was calculated to quantify the effect sizes [31, 32]. This was conducted on baseline characteristics with a mean difference and a clinical significance that can have an impact on the outcome, as multiple comparisons may increase the risk of type I error (false positive). These variables were age at treatment start, HSDS for bone age, and IGF-I SDS. For children who reached NAH, the statistical difference in HSDS was calculated.

Sensitivity analyses were carried out to validate the primary effectiveness data. As diagnoses of ISS and GHD are dependent on local guidance and evolve over time, sensitivity analyses were conducted using GH peak data with cutoffs at ≥7 ng/mL and <7 ng/mL, and at ≥10 ng/mL and <10 ng/mL to define ISS and GHD, respectively [33-35]. Further sensitivity analyses considered mean age of treatment initiation and mean duration of treatment.

## Results

### Study Population

The SAS included 26 674 children, of whom 3816 had a diagnosis of ISS and 22 858 had a diagnosis of GHD, as reported by their physician (Fig. 1). Of these children, 18 405 were included in the EAS (ISS: 2684; GHD: 15 721) and 1856 in the NAH population (ISS: 230; GHD: 1626). Of the total GHD diagnoses in the EAS, 12 490 (79.5%) and 3231 (20.6%) were deemed nonidiopathic GHD and idiopathic GHD, respectively. Baseline clinical characteristics of the EAS are presented in Table 1 and Table S1 [36]. The ISS group of the EAS started GH treatment at a mean (SD) age of 11.2 (3.2) years, which was later than the GHD group (10.2 [4.0] years). Mean (SD) difference in age at treatment start between the 2 groups was 1.03 (3.87, *P* < .0001, Cohen's d = 0.26). Mean (SD) difference in HSDS for bone age between the 2 groups was −0.52 (1.47, *P* < .0001, Cohen's d = −0.35). Mean (SD) difference in IGF-I SDS between the 2 groups was 0.48 (1.70, *P* < .0001, Cohen's d = 0.28).

**Table 1.** Baseline characteristics of children with GHD and ISS in the EAS

	ISS (n = 2684)	GHD (n = 15 721)	Mean difference (SD) *P* value (Cohen's d statistic)
Male/female, n (%)	1813 (68)/871 (32)	11 066 (70)/4655 (30)	
Age at treatment start (year)	11.22 (3.24)	10.19 (3.97)	1.03 (3.9) *P* < .0001 (0.27)
BA/CA	0.89 (0.14)	0.84 (0.19)	
HSDS	−2.28 (0.89)	−2.24 (1.08)	
HSDS for bone age	−1.16 (1.15)	−0.64 (1.52)	−0.52 (1.5) *P* < .0001 (−0.35)
Target HSDS	−0.71 (0.93)	−0.52 (0.99)	
Target HSDS − HSDS	1.56 (1.15)	1.72 (1.26)	
GH peak (ng/mL)	15.64 (9.96)	5.44 (4.83)	
IGF-I SDS	−1.13 (1.7)	−1.61 (1.69)	0.48 (1.7) *P* < .0001 (0.28)
IGFBP-3 SDS	−1.00 (2.17)	−1.24 (1.83)	
Dose at baseline (mg/kg/day)	0.05 (0.01)	0.04 (0.01)	
GH treatment duration (year)	2.31 (2.03)	3.08 (2.54)	
Gestational age (weeks)	38.88 (2.65)	38.71 (2.74)	
Birth weight (g)	3020 (656)	3070 (682)	
Birth weight SDS	−0.50 (1.23)	−0.33 (1.25)	
Birth length (cm)	48.91 (3.42)	49.1 (3.79)	
Birth length SDS	−0.73 (1.50)	−0.51 (1.55)	
Height velocity (cm/year)	5.16 (2.08)	5.58 (2.86)	
HVSDS	−0.38 (1.67)	−0.61 (1.89)	

Data are mean (SD) unless otherwise stated. IGF-1 and IGFBP-3 measurements were derived from local assays.

Abbreviations: BA/CA, bone age to chronological age ratio; EAS, effectiveness analysis set; HD, GH deficiency; HSDS, height SD score; HVSDS, height velocity SD score; IGFBP-3, insulin-like factor binding protein-3; ISS, idiopathic short stature; N, number of children; SDS, SD score.

Children who reached NAH were also older at treatment start (ISS: 13.4 [2.5] years; GHD: 12.9 [3.2] years) (for data per sex see Table S2 [36]). In the NAH population, female children with ISS and GHD started puberty at a similar age (ISS: 12.1 [1.9] years; GHD: 12.2 [2.0] years), as did male children (ISS: 13.7 [1.7] years; GHD: 13.9 [1.9] years). Baseline characteristics for children with and without baseline GH peak data were comparable (Table S3) [36].

### GH Exposure

**Figure 2.**
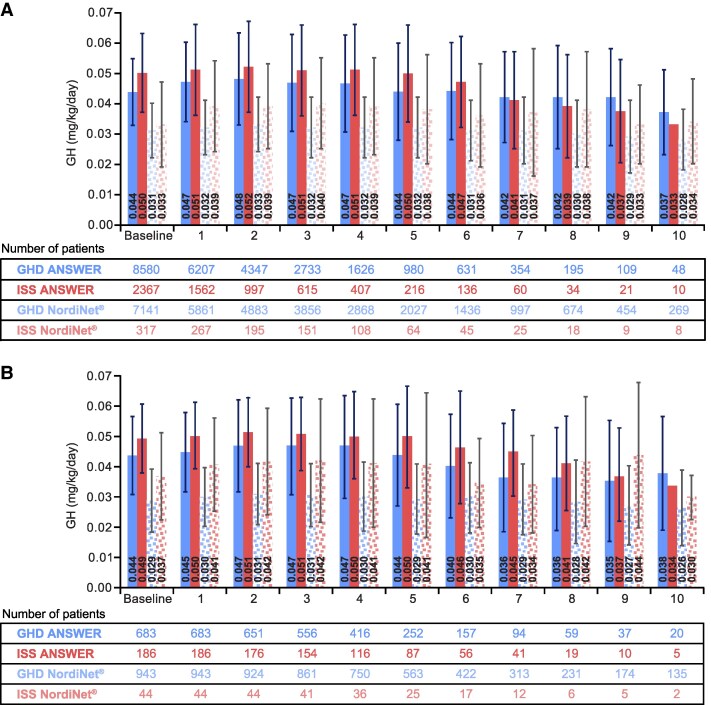
Average GH dose exposure for children with ISS and GHD. (A) EAS (SD); (B) NAH. Data are mean (SD). Abbreviations: EAS, effectiveness analysis set; GHD, GH deficiency; ISS, idiopathic short stature; NAH, near adult height.

Throughout the 10 years of follow-up, mean (SD) GH dose exposure for children with ISS was consistently higher than for children with GHD (Fig. 2). The number of children treated with GH in the EAS decreased from baseline (ISS: 2684; GHD: 15 721) to 5 (ISS: 280; GHD: 3007) and 10 years (ISS: 18; GHD: 317) in both groups. Mean (SD) GH dose across the 10 years of treatment was 25% higher in children with ISS (0.050 [0.014] mg/kg/day) compared with children with GHD (0.040 [0.013] mg/kg/day). Mean [SD] duration of treatment was 34% longer for children with GHD (3.082 [2.544] years) than for those with ISS (2.307 [2.030] years).

There were fewer children in the NAH population, decreasing from baseline (ISS: 230; GHD: 1626) to 5 (ISS: 112; GHD: 815) and 10 years (ISS: 7; GHD: 155) in both groups. Mean (SD) GH doses in the NAH population were similar to in the EAS (ISS: 0.049 [0.013] mg/kg/day; GHD: 0.036 [0.014] mg/kg/day), whereas mean [SD] duration of treatment was longer (ISS: 4.16 [2.20] years; GHD: 4.68 [2.85] years).

### Effectiveness

#### Growth response in EAS

**Figure 3.**
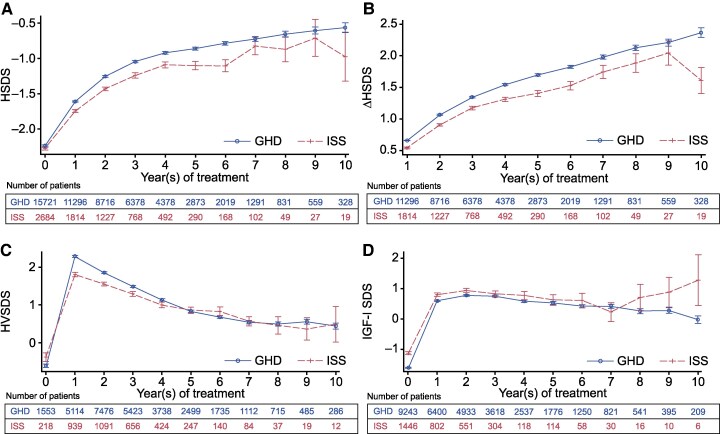
Growth outcomes from baseline to 10 years in children with GHD and ISS. (A) Height SDS; (B) change in height SDS; (C) height velocity SDS; (D) IGF-I SDS. Data are mean (StdErr). Abbreviations: EAS, effectiveness analysis set; GHD, GH deficiency; HSDS, height SDS; ΔHSDS, change in HSDS; HVSDS, height velocity SDS; ISS, idiopathic short stature; SDS, SD score; StdErr, standard error. IGF-1 measurements were derived from local assays.

GH treatment was associated with increase in mean [SD] HSDS from baseline (ISS: −2.28 [0.89]; GHD: −2.24 [1.08]) to 5 (ISS: −1.10 [1.01]; GHD: −0.86 [1.11]) and 10 years (ISS: −0.98 [1.52]; GHD: −0.56 [1.24]) in both groups (Fig. 3A). Distribution of HSDS levels progressed from most children having a HSDS <−2 at baseline, to most children having HSDS ±2 after 1 year and throughout follow-up for both groups (Table 2). As with HSDS, mean [SD] ΔHSDS increased over follow-up in both groups from 1 year (ISS: 0.54 [0.43]; GHD: 0.66 [0.54]) through to 5 years (ISS: 1.40 [0.81]; GHD: 1.69 [0.10]) (Fig. 3B). In the ISS group, variability in growth response was observed after 6 years of treatment; mean (SD) ΔHSDS peaked at 2.04 (1.00) at 9 years and declined to 1.61 (0.89) at 10 years. Conversely, a continual increase until 10 years (mean 2.34 [SD 1.36]) was observed in the GHD group. Trends in HSDS and ΔHSDS from baseline throughout follow-up were similar in children with ISS and GHD, whether diagnosis was physician-determined or defined using GH peak data (Fig. S2) [36].

**Table 2.** Distribution of HSDS and IGF-I SDS levels by year of treatment

	GHD			ISS		
	SDS < −2	SDS ±2	SDS ≥2	SDS <−2	SDS ±2	SDS ≥2
**Height SDS, % of participants**						
Baseline	60.66	39.26	0.08	65.91	34.09	0.00
1 year	32.68	67.09	0.24	35.28	64.72	0.00
2 years	21.05	78.65	0.30	24.12	75.63	0.24
3 years	15.66	83.74	0.60	20.18	79.56	0.26
4 years	13.80	85.52	0.69	14.02	85.98	0.00
5 years	13.44	85.76	0.80	15.52	84.48	0.00
6 years	12.78	86.23	0.99	17.26	82.74	0.00
7 years	12.08	86.52	1.39	14.71	84.31	0.98
8 years	12.27	86.16	1.56	12.24	87.76	0.00
9 years	10.20	88.91	0.89	11.11	85.19	3.70
10 years	14.33	83.84	1.83	21.05	78.95	0.00
**IGF-I SDS, % of participants**						
Baseline	42.39	54.58	3.03	32.37	62.66	4.98
1 year	5.95	74.03	20.02	4.61	72.69	22.69
2 years	5.31	71.86	22.83	3.45	68.42	28.13
3 years	6.25	70.90	22.86	4.93	71.71	23.36
4 years	6.62	73.28	20.10	5.85	68.62	25.53
5 years	6.70	73.48	19.82	8.77	70.18	21.05
6 years	8.80	72.40	18.80	10.34	65.52	24.14
7 years	8.28	73.57	18.15	10.00	76.67	13.33
8 years	10.91	70.98	18.11	12.50	68.75	18.75
9 years	9.37	75.19	15.44	0.00	60.00	40.00
10 years	12.44	76.08	11.48	0.00	50.00	40.00

IGF-1 measurements were derived from local assays.

Abbreviations: GHD, GH deficiency; HSDS, height SD score; ISS, idiopathic short stature; SDS, SD score.

There was an increase in mean [SD] HVSDS from baseline to 1 year in children with ISS (−0.38 [1.67] to 1.80 [1.68]) and children with GHD (−0.61 [1.89], 2.29 [1.92]); levels remained >0 for the remainder of follow-up (Fig. 3C). IGF-I SDS (mean [SD]) increased from baseline to 1 year for both groups (ISS: −1.13 [1.71] to 0.80 [1.63]; GHD: −1.61 [1.69] to 0.60 [1.69]), remaining within the normal range (SDS ±2) throughout follow-up (Fig. 3D). Few children in both groups had IGF-I levels SDS <−2 after 1 year (<6%) and throughout follow-up (Table 2).

### Near Adult Height

Evolution of change in HSDS, IGF-SDS, and HVSDS in the NAH population is shown in Fig. S3 [36]. Baseline HSDS (mean [SD]) in the NAH population was −2.43 (0.91) for children with ISS (target HSDS of −0.64) and −2.36 (1.16) for children with GHD (target HSDS of −0.50). At NAH, mean (SD) HSDS was −1.21 (1.09) and −0.90 (1.20) for children with ISS and GHD, respectively. Mean (SD) difference in HSDS between the 2 groups was −0.31 (1.2, *P* < .0001, Cohen's d = −0.26).

Mean (SD) ΔHSDS from baseline to 10 years was 1.21 (0.86) and 1.45 (1.09). Distribution of HSDS progressed from most children in the NAH group having a HSDS <−2 at baseline, to most children having HSDS ±2 after 1 year and throughout follow-up for both ISS and GHD groups (Table S4) [36]. Distribution of IGF-I SDS levels followed a similar pattern to HSDS for the GHD group, but for those with ISS, most children had IGF-I levels SDS ±2 at baseline (56.5%) and throughout follow-up. The only exception to this distribution occurred at 9 years, where the majority (75.0%) of children with ISS had IGF-I levels SDS ≥2 (Table S4) [36].

### BA/CA

BA/CA (mean [SD]) for both ISS and GHD groups increased from baseline (0.89 [0.11] and 0.84 [0.14], respectively) but did not exceed 1 throughout the 10-year follow-up.

### Effect of Age of Treatment Initiation and Treatment Duration on Growth Response

Children with ISS with ≥8 years’ follow-up reached height SDS within the normal range (SDS ±2). Comparatively, children with shorter treatment duration were more likely to have only reached height SDS <−2. In a sensitivity analysis of patients categorized by age of treatment start, children with ISS who started treatment before 3 years of age achieved a higher HSDS than those who initiated treatment at a later age, across all years of treatment (Fig. S4A) [36]. At year 8, the mean (SD) HSDS for children initiating treatment before 3 years of age was 0.1 (1.0), compared with −0.7 (1.4), −1.5 (1.2), and −0.9 (0.8) for those aged 3 to 6, 6 to 9, and >9 years, respectively. Between groups of patients who started treatment after 3 years of age (3-6, 6-9, and >9 years of age), no relevant difference was observed. Overall, children with GHD started treatment much earlier than children with ISS, with a greater proportion starting before 7 years old (Fig. S4B and S4C) [36].

### Safety

The overall rates and frequencies of ARs across the 10-year follow-up were similar between groups. Total PYEs from which IRs were calculated were 9186.4 and 72 274.2 for ISS and GHD patients, respectively. The IR per 1000 PYE of overall ARs was 7.18 for children with ISS and 6.96 for children with GHD (IRR: 1.03; 95% CI, .50-2.12), the majority of which were NSARs (IR: 6.20 vs 5.23, respectively; IRR: 1.19; 95% CI, .61-2.30). For children with SARs, the overall IR was 0.98 and 1.73 for children with ISS and GHD, respectively (IRR: 0.57; 95% CI, .03-12.17). Across the 10-year follow-up, the frequencies of ARs/NSARs/SARs in the ISS and GHD groups followed similar patterns by the Medical Dictionary for Regulatory Activities system organ classes (Fig. S5) [36]. The most common types of disorders for ARs were muscular and connective tissue disorders (IR 2.94 for ISS and 2.05 for GHD), nervous system disorders (IR 2.18 for ISS and 2.01 for GHD), and general disorders and administration-site conditions (IR 0.76 for ISS and 1.08 for GHD). For these conditions, the IR of SARs was 0.54, 0.11, and 0 for children with ISS, and 0.40, 0.35, and 0.15 for children with GHD, respectively. Neoplasms benign, malignant, and unspecified (including cysts and polyps) were reported in 1 child with ISS (IR: SAR, 0.11) and 20 children with GHD (IR: SAR, 0.24; NSAR, 0.06). No deaths were reported among children with ISS and 8 deaths were reported among children with GHD; of these, 7 were deemed unlikely to be related to treatment. The death of 1 child from leptomeningeal melanocytosis, who had a medical history comprising supracellular cyst, hydrocephalus, and congenital neurocutaneous melanosis, and was 7 years old at start of treatment and 16 years old on AR onset, was possibly related to treatment, according to investigator assessment.

## Discussion

In this post hoc analysis, GH treatment effectively improved height outcomes in children with ISS and GHD over long-term follow-up, with IGF-I mean levels remaining within normal range and no observation of inappropriate advancement of BA/CA. These results included individuals who achieved NAH, where NAH was closer to target HSDS than baseline HSDS. The safety profile of GH treatment was tolerable and comparable in children with ISS and GHD.

Average GH dose for children with ISS was higher than for those with GHD across all treatment years, suggesting that children with ISS are less responsive to treatment because they are not deficient in endogenous GH [5]. Children in the United States received greater doses of GH across all years of treatment compared with those in Europe, highlighting regional prescribing differences. Likewise, initial treatment doses in the Kabi/Pfizer International Growth Database (KIGS) data, which included information from children with ISS, GHD, and others who were treated with recombinant human GH, were also higher in the United States [20].

Mean duration of treatment was longer for children with GHD compared to children with ISS, and children with GHD, on average, started treatment a year earlier. This is likely influenced by late diagnosis of ISS and insurance rejections for treatment for these patients (particularly in the United States). Indeed, the difference in age at treatment start between the 2 groups was statistically significant and showed a small effect size (Cohen's d = 0.26). Earlier GH treatment start has been shown to be a strong predictor of greater height outcomes [37, 38]. In the present analysis, children with ISS who started treatment before 3 years of age exhibited superior catch-up growth and greater improvement in HSDS compared with those who started treatment later. In addition, persistence of GH treatment of children with ISS for ≥8 years was associated with a greater improvement in height outcomes compared with children who had shorter treatment durations. Children who achieved NAH were approximately 2 years older at treatment start than those who had not yet completed growth; thus, it is possible that effectiveness results pertaining to the NAH population could underestimate the full potential of GH treatment. In addition, children with ISS had a shorter duration of GH treatment because they started treatment at an older age, which resulted in lesser height gains despite receiving a higher dose of GH. Nevertheless, these observations highlight the importance of early identification and early and continued GH treatment in children with ISS for the attainment of NAH, as well as height attainment in line with their genetic potential.

Trends in HSDS, ΔHSDS, and HVSDS from baseline to 10 years were similar between children with ISS and children with GHD, although outcomes were slightly greater for children with GHD. Although the difference in HSDS for children who reached NAH was statistically significant between the 2 groups, the effect size was small (Cohen's d = −0.26). Several anthropometric measures (weight, initial height, body mass index) and IGF-I have been shown to be predictive factors for growth in children with GHD [39]. Although the effect of these parameters was not investigated, the results of the present analysis align with previous studies comparing the effects of GH treatment in children with ISS and GHD, where similar effects on short-term growth response between groups were observed [37, 40-42]. Specifically, analysis of data from KIGS demonstrated comparable ΔHSDS among children with ISS and congenital GHD in the first year of treatment. Consistent with the present study, GH response in prepubertal children with GHD was slightly more favorable than in those with ISS (ΔHSDS for GHD: 1.01; ISS: 0.55), as was the response in pubertal children, albeit smaller (ΔHSDS for GHD: 0.49; ISS: 0.39) [20]. Furthermore, in this study, treatment with GH was effective in the NAH population, with mean ΔHSDS >1 in both groups throughout follow-up. Comparatively, analysis of data from KIGS showed median gains in NAH SDS were >1 in children with ISS and GHD across follow-up [20]. In a systematic review, GH treatment was shown to significantly improve adult height of children with ISS vs untreated controls, with a ΔHSDS of 1.06 (0.30) and 0.18 (0.27), respectively (*P* < .001) [8]. This effect was enhanced in children who received higher doses of GH treatment vs lower doses [8]. Similar results were demonstrated following a meta-analysis of 21 studies investigating the use of GH in children with ISS [7]. Children who received recombinant human GH exhibited significantly higher height increment compared with untreated controls after 1 year and, in longer-term studies, after 2 years of treatment. The effect of GH on adult height attainment was also analyzed, demonstrating that treated children experienced significantly increased adult height compared with controls [7]. The present study builds on previous studies of the use of GH in children with ISS and GHD, offering evidence of the beneficial effects of GH on height outcomes for up to 10 years of treatment.

The difference in mean IGF-I SDS at baseline was statistically significant between the 2 groups but, the effect size was small (Cohen's d = 0.28). Importantly, mean (SD) IGF-I SDS increased >0 in the first year of follow-up (ISS: 0.80 [1.63]; GHD: 0.60 [1.69]) and did not drop below this afterwards across both indications. In addition, the number of children who exceeded the normal range of IGF-I was minimal. Children diagnosed with ISS may have varying degrees of IGF-I deficiency and, although this is usually less marked than in severe GHD or classical IGF-I deficiency, some children have IGF-I levels below −2 SDs of normal [43]. Children with lower IGF-I levels pretreatment can have significantly lower HSDS at baseline compared with those with normal IGF-I levels [44]. However, this can lead to greater increase in HSDS after 1 year of GH treatment.

Across all effectiveness outcomes, variability in response was observed in later years of treatment in children with ISS. The observed plateau in GH response among children with ISS between years 4 and 6 of treatment indicates that some, but not all, of the children with ISS may have ceased to respond to GH. In addition, those with ISS initiating treatment aged older than 3 years showed little height gain after 4 years of treatment. The variability in growth response observed in children with ISS after 6 years of treatment in this study may be explained by the differing responses to GH documented in each etiology of ISS [45], and the lack of response among older children initiating treatment could be due to the adverse impact of delayed treatment start on height outcomes [37, 38]. However, because HVSDS did not drop below 0 either in children with ISS or GHD, it could be considered as a rationale for continuing treatment in children gaining HSDS within these populations. For children with ISS specifically, continuing GH treatment for 6 years at doses of 0.37 mg/kg/week has been shown to significantly increase height velocity compared with lower doses, with 94% of children reaching NAH [46]. The results of the present analysis provide a reasonable basis for continued treatment and appropriate dose escalation in children with ISS who respond to GH and are in line with previous guidance recommending that GH treatment in this population should continue until NAH attainment [5].

The safety profile of GH treatment was tolerable and similar across both indications. The incidence rate of ARs was low with few SARs, and no new safety signals were observed. Notably, IR of neoplasms was low for both groups. Although this has previously been regarded as an area of concern, there is insufficient clinical evidence in the present study to support an association of neoplasms or increased risk of cancer recurrence in patients with GH treatment [47].

This study was strengthened by using data from 2 large, international, observational studies. Children were followed over a long period in a real-world setting, which offered an inclusive picture of the use and effectiveness of GH treatment in clinical practice. Furthermore, the large population allowed for investigation of subgroups and infrequent events and strengthened the statistical power of the data.

As this was a longitudinal, observational study, changes in diagnostic and eligibility guidelines for GH treatment may have occurred during the study. There was also potential for confounding of results by local differences in diagnostics, laboratory analyses, and events reporting. In addition, the incorporation of multiple sites with distinct cultures and approaches in the management and ascertainment of variables used in this analysis, as well as selection biases relating to access to medication and physician-determined inclusion in the NordiNet IOS and ANSWER Program study, may have resulted in inconsistent data collection. Other limitations included potential underreporting of safety outcomes and lack of an untreated control group. Furthermore, the low number of children diagnosed with ISS, and hence included in this analysis compared with GHD, resulted in a depleted sample size, particularly after 6 years of follow-up. This may be due to misclassification of patients by physicians before inclusion in the study, exclusion of patients who did not meet all criteria for inclusion (no valid GH exposure information, no valid mean GH dose, invalid birthdate, or initiation of treatment after 18 years of age and last measurement before 20 years of age), or discontinuation of treatment from poor response after 6 years of treatment. Another possible reason is that Norditropin is only indicated for ISS in the United States and South Korea [26, 28], whereas it is approved for GHD in Europe, the United States, and Japan [25-27]; hence, the available treated patients with ISS are likely to be more restricted.

A specific limitation was incomplete reporting of variables, particularly GH peak test results. Because of this, children were assigned to the ISS or GHD groups based on physician-reported diagnosis. Criteria for diagnosis of these conditions can vary between countries, and it is also possible for children to be misdiagnosed; both factors may have led to incorrect grouping. Additionally, the GH provocative tests used to confirm GHD diagnosis and their recommended cutoffs differ between countries [48]. GH secretion significantly varies in the early years of development and those leading up to puberty [49]. High false-positive rates of GHD diagnosis through provocative testing have been identified in pre- and peripubertal children [50], and many children with idiopathic GHD (comprising >20% of children in this analysis) exhibit GH responses that imply a reversal of GHD after postpubertal retesting [51]. In the present study, the authors did not investigate the persistence of GHD in pubertal children at the end of GH treatment, nor was HSDS adjusted for pubertal status; however, sensitivity analyses of effectiveness data from children grouped by GH peak cutoff (7 and 10 ng/mL) confirmed that height outcomes did not differ regardless of how children were grouped. The present analysis also did not account for the influence of GHD etiology on GH response, nor did it investigate how differences in patient and parent perceptions of short stature vary between sexes, the latter being an important consideration when discussing and diagnosing ISS in a clinical setting [52]. Nevertheless, sharing the results from comprehensive databases like Nordinet IOS and ANSWER in studies such as the one described here enhances understanding of the impact of GH treatment on growth patterns in a broader population.

## Conclusions

This was the first analysis to compare the long-term effectiveness and safety of GH treatment in children diagnosed with ISS and GHD. Results from the NordiNet IOS and the ANSWER Program show that GH treatment effectively increases height in children with ISS for up to 10 years, including those who reached NAH. The safety profile of Norditropin observed in patients with ISS was comparable to those with GHD, supporting a favorable benefit–risk profile of GH treatment in children diagnosed with ISS. These findings provide useful information on how and when to treat children with ISS and highlight the need for early and continued GH treatment in this population for attainment of NAH. However, further studies are needed to define a more homogeneous ISS population for the evaluation of GHT.

## Data Availability

Data collected for the study will be shared in data sets in a deidentified/anonymized format. Study protocol and redacted clinical study report will be available according to Novo Nordisk data-sharing commitments. The data will be available via access request proposal form and the access criteria can be found at novonordisk-trials.com. The data will be made available on a specialized SAS data platform. Data will be shared with bona fide researchers and for use as approved by the independent review board according to the IRB Charter.

## Abbreviations

ΔHSDS: change in height SD score
ANSWER: American Norditropin Studies: Web-Enabled Research
AR: adverse reaction
BA/CA: bone age/chronological age ratio
EAS: effectiveness analysis set
GHD: GH deficiency
HSDS: height SD score
HVSDS: height velocity SD score
IR: incidence rate
IRR: incidence rate ratio
ISS: idiopathic short stature
KIGS: Kabi/Pfizer International Growth Database
NAH: near adult height
NSAR: nonserious adverse reaction
PYE: patient-years of exposure
SAR: serious adverse reaction
SAS: safety analysis set
SDS: SD score

## References

[1] Wit JM, Clayton PE, Rogol AD, Savage MO, Saenger PH, Cohen P. Idiopathic short stature: definition, epidemiology, and diagnostic evaluation. Growth Horm IGF Res. 2008;18(2):89‐110. 18182313 10.1016/j.ghir.2007.11.004

[2] Yau M, Lu J, Rapaport R, et al Idiopathic short stature and growth failure of unknown etiology. In: Feingold KR, ed. Endotext. MDText.com, Inc.; 2000. 37910635

[3] Cohen LE, Rogol AD. Children with idiopathic short stature: an expanding role for genetic investigation in their medical evaluation. Endocr Pract. 2024;30(7):679‐686. 38679385 10.1016/j.eprac.2024.04.009

[4] Wit JM, Joustra SD. Long-acting PEGylated growth hormone in children with idiopathic short stature: time to reconsider our diagnostic and treatment policy? Eur J Endocrinol. 2023;188(1):lvac005. 36651155 10.1093/ejendo/lvac005

[5] Cohen P, Rogol AD, Deal CL, et al Consensus statement on the diagnosis and treatment of children with idiopathic short stature: a summary of the Growth Hormone Research Society, the Lawson Wilkins Pediatric Endocrine Society, and the European Society for Paediatric Endocrinology Workshop. J Clin Endocrinol Metab. 2008;93(11):4210‐4217. 18782877 10.1210/jc.2008-0509

[6] Bryant J, Baxter L, Cave CB, Milne R. Recombinant growth hormone for idiopathic short stature in children and adolescents. Cochrane Database Syst Rev. 2007;3:Cd004440. 10.1002/14651858.CD004440.pub2 17636758

[7] Paltoglou G, Dimitropoulos I, Kourlaba G, Charmandari E. The effect of treatment with recombinant human growth hormone (rhGH) on linear growth and adult height in children with idiopathic short stature (ISS): a systematic review and meta-analysis. J Pediatr Endocrinol Metab. 2020;33(12):1577‐1588. 33035189 10.1515/jpem-2020-0287

[8] Muammar T, Alhasaeri M. A systematic review of the impact of growth hormone therapy on final adult height of children with idiopathic short stature. J Diabetes Endocrine Practice. 2024;07(01):25‐34.

[9] Grimberg A, DiVall SA, Polychronakos C, et al Guidelines for growth hormone and insulin-like growth factor-I treatment in children and adolescents: growth hormone deficiency, idiopathic short stature, and primary insulin-like growth factor-I deficiency. Horm Res Paediatr. 2016;86(6):361‐397. 27884013 10.1159/000452150

[10] Bullinger M, Bloemeke J, Mericq V, et al Quality of life in adolescent boys with idiopathic short stature: positive impact of growth hormone and aromatase inhibitors. Horm Res Paediatr. 2019;90(6):381‐392. 10.1159/000496353 30820008

[11] Cardoso DF, Martinelli Jr. CE, Campos VC, et al Comparison between the growth response to growth hormone (GH) therapy in children with partial GH insensitivity or mild GH deficiency. Arq Bras Endocrinol Metabol. 2014;58(1):23‐29. 24728160 10.1590/0004-2730000002793

[12] Chaplin JE, Kriström B, Jonsson B, et al Improvements in behaviour and self-esteem following growth hormone treatment in short prepubertal children. Horm Res Paediatr. 2011;75(4):291‐303. 21304250 10.1159/000322937

[13] Ross JL, Sandberg DE, Rose SR, et al Psychological adaptation in children with idiopathic short stature treated with growth hormone or placebo. J Clin Endocrinol Metab. 2004;89(10):4873‐4878. 15472178 10.1210/jc.2004-0791

[14] Sandberg DE . Quality of life and self-esteem in children treated for idiopathic short stature. J Pediatr. 2003;143(5):691. 14649665 10.1067/S0022-3476(03)00395-0

[15] Pedicelli S, Peschiaroli E, Violi E, Cianfarani S. Controversies in the definition and treatment of idiopathic short stature (ISS). J Clin Res Pediatr Endocrinol. 2009;1(3):105‐115. 21274395 10.4008/jcrpe.v1i3.53 PMC3005647

[16] Hintz RL . Growth hormone treatment of idiopathic short stature. Horm Res. 1996;46(4-5):208‐214. 8950623 10.1159/000185025

[17] Ranke MB, Lindberg A, Mullis PE, et al Towards optimal treatment with growth hormone in short children and adolescents: evidence and theses. Horm Res Paediatr. 2013;79(2):51‐67. 23446062 10.1159/000347121

[18] Grumbach MM, Bin-Abbas BS, Kaplan SL. The growth hormone cascade: progress and long-term results of growth hormone treatment in growth hormone deficiency. Horm Res. 1998;49(Suppl 2):41‐57. 9716827

[19] Sävendahl L, Battelino T, Højby Rasmussen M, Brod M, Saenger P, Horikawa R. Effective GH replacement with once-weekly somapacitan vs daily GH in children with GHD: 3-year results from REAL 3. J Clin Endocrinol Metab. 2022;107(5):1357‐1367. 34964458 10.1210/clinem/dgab928 PMC9016428

[20] Maghnie M, Ranke MB, Geffner ME, et al Safety and efficacy of pediatric growth hormone therapy: results from the full KIGS cohort. J Clin Endocrinol Metab. 2022;107(12):3287‐3301. 36102184 10.1210/clinem/dgac517 PMC9693805

[21] Ariza-Jimenez A-B, Leiva Gea I, Martinez-Aedo Ollero MJ, Lopez-Siguero JP. Isolated growth hormone deficiency and idiopathic short stature: comparative efficiency after growth hormone treatment up to adult height. J Clin Med. 2021;10(21):4988. 34768508 10.3390/jcm10214988 PMC8585059

[22] Gad AA, Shamma R, Elmonem MA, Badawi NE, Fawaz L, Hassan MM. Growth hormone therapy response in children with short stature. Egyptian Pediatric Association Gazette. 2023;71(1):27.

[23] Ma Y, Sheng J, Wang L, Zhang Y, Liu L. Therapeutic efficacy of recombinant human growth hormone in children with different etiologies of dwarfism from a pharmacoeconomic point of view. Medicine (Baltimore). 2024;103(25):e38350. 38905369 10.1097/MD.0000000000038350 PMC11191898

[24] Backeljauw P, Miller BS, Levy R, et al PATRO children, a multi-center, non-interventional study of the safety and effectiveness of omnitrope(®) (somatropin) treatment in children: update on the United States cohort. J Pediatr Endocrinol Metab. 2021;34(4):431‐440. 33647196 10.1515/jpem-2020-0360

[25] Electronic Medicines Compendium 2022. Norditropin FlexPro 5 mg/1.5 mL. 2022 18 December 2024]; Available from: https://www.medicines.org.uk/emc/product/11755/smpc

[26] Food and Drug Administration 2017. Norditropin Prescribing Information. 2017 18 December 2024]; Available from: https://www.accessdata.fda.gov/drugsatfda_docs/label/2020/021148s053lbl.pdf

[27] Pharmaceuticals and Medical Devices Agency 2022. Report on investigation results. 2022 14 December 2024]; Available from: https://www.pmda.go.jp/files/000246397.pdf

[28] Chae HW, Hwang I-T, Lee J-E, et al Height outcomes in Korean children with idiopathic short stature receiving growth hormone treatment. Front Endocrinol (Lausanne). 2022;13:925102. 36157444 10.3389/fendo.2022.925102 PMC9490583

[29] Höybye C, Sävendahl L, Christesen HT, et al The NordiNet® international outcome study and NovoNet® ANSWER program®: rationale, design, and methodology of two international pharmacoepidemiological registry-based studies monitoring long-term clinical and safety outcomes of growth hormone therapy (norditropin®). Clin Epidemiol. 2013;5:119‐127. 23658497 10.2147/CLEP.S42602 PMC3641810

[30] Sävendahl L, Polak M, Backeljauw P, et al Treatment of children with GH in the United States and Europe: long-term follow-up from NordiNet® IOS and ANSWER program. J Clin Endocrinol Metab. 2019;104(10):4730‐4742. 31305924 10.1210/jc.2019-00775 PMC6812718

[31] Sullivan GM, Feinn R. Using effect size-or why the P value is not enough. J Grad Med Educ. 2012;4(3):279‐282. 23997866 10.4300/JGME-D-12-00156.1 PMC3444174

[32] Goulet-Pelletier J-C, Cousineau D. A review of effect sizes and their confidence intervals, part I: the Cohen's d family. Quant Method Psychol. 2018;14(4):242‐265.

[33] Collett-Solberg P, Ambler G, Backeljauw P, et al Diagnosis, genetics, and therapy of short stature in children: a growth hormone research society international perspective. Horm Res Paediatr. 2019;92(1):1‐14. 10.1159/000502231 PMC6979443 31514194

[34] Growth Hormone Research Society . Consensus guidelines for the diagnosis and treatment of growth hormone (GH) deficiency in childhood and adolescence: summary statement of the GH Research Society. J Clin Endocrinol Metab. 2000;85(11):3990‐3993. 11095419 10.1210/jcem.85.11.6984

[35] Donbaloğlu Z, Singin B, Acar S, et al Evaluation of the growth response of children with growth hormone deficiency according to the peak growth hormone levels in provocation tests. Archives de Pédiatrie. 2023;30(8):573‐579. 37802668 10.1016/j.arcped.2023.08.005

[36] Phillip M, Abuzzahab M J, Pietropoli A, et al Supplementary material for “Comparative Outcomes of GH Treatment in Pediatric Idiopathic Short Stature and GH Deficiency”. 2025. Available from: 10.6084/m9.figshare.30042241. Date of deposit 03 September 2025. PMC12448476 40980545

[37] Jeong H, Kwon E, Shim Y, Lee H, Hwang J. Comparative study of growth hormone treatment in children with idiopathic short stature and growth hormone deficiency. Curr Drug Metab. 2015;16(10):940‐946. 26467072 10.2174/1389200216666151015112841

[38] Dauber A, Phillip M, Ferran J-M, et al Clinical predictors of good/poor response to growth hormone treatment in children with idiopathic short stature. Horm Res Paediatr. 2024:1‐12. 10.1159/000542579 PMC13089979 39571543

[39] Mastromauro C, Bin-Abbas BS, Kaplan SL. Predictive factors of adult height after 2 years of GH replacement therapy in children with growth hormone deficiency (GHD). Horm Res Paediatr. 2022;95(Suppl 2):1‐616.

[40] Hou L, Liang Y, Wu W, Lin H-H, Luo X-P, Ying Y-Q. Comparison of the efficacy and safety of recombinant human growth hormone in treating idiopathic short stature and growth hormone deficiency in children. Growth Horm IGF Res. 2020;53–54:101331. 10.1016/j.ghir.2020.101331 32777706

[41] Yoon JY, Cheon CK, Lee JH, et al Response to growth hormone according to provocation test results in idiopathic short stature and idiopathic growth hormone deficiency. Ann Pediatr Endocrinol Metab. 2022;27(1):37‐43. 35038835 10.6065/apem.2142110.055 PMC8984754

[42] Child CJ, Quigley CA, Cutler GB Jr, et al Height gain and safety outcomes in growth hormone-treated children with idiopathic short stature: experience from a prospective observational study. Horm Res Paediatr. 2019;91(4):241‐251. 31185471 10.1159/000500087

[43] Şıklar Z, Kocaay P, Çamtosun E, et al The effect of recombinant growth hormone treatment in children with idiopathic short stature and low insulin-like growth factor-1 levels. J Clin Res Pediatr Endocrinol. 2015;7(4):301‐306. 26777041 10.4274/jcrpe.2111 PMC4805225

[44] Soliman A, Rogol AD, Elsiddig S, et al Growth response to growth hormone (GH) treatment in children with GH deficiency (GHD) and those with idiopathic short stature (ISS) based on their pretreatment insulin-like growth factor 1 (IGFI) levels and at diagnosis and IGFI increment on treatment. J Pediatr Endocrinol Metab. 2021;34(10):1263‐1271. 34291621 10.1515/jpem-2021-0389

[45] Savage MO, Storr HL. GH resistance is a component of idiopathic short stature: implications for rhGH therapy. Front Endocrinol (Lausanne). 2021;12:781044. 34956092 10.3389/fendo.2021.781044 PMC8702638

[46] Wit JM, Rekers-Mombarg LTM, Cutler GB, et al Growth hormone (GH) treatment to final height in children with idiopathic short stature: evidence for a dose effect. J Pediatr. 2005;146(1):45‐53. 15644821 10.1016/j.jpeds.2004.08.055

[47] Wilton P, Mattsson AF, Darendeliler F. Growth hormone treatment in children is not associated with an increase in the incidence of cancer: experience from KIGS (Pfizer International Growth Database). J Pediatr. 2010;157(2):265‐270. 20400105 10.1016/j.jpeds.2010.02.028

[48] Binder G, Reinehr T, Ibáñez L, et al GHD diagnostics in Europe and the US: an audit of national guidelines and practice. Horm Res Paediatr. 2019;92(3):150‐156. 31707392 10.1159/000503783

[49] Yuen KCJ, Johannsson G, Ho KKY, Miller BS, Bergada I, Rogol AD. Diagnosis and testing for growth hormone deficiency across the ages: a global view of the accuracy, caveats, and cut-offs for diagnosis. Endocr Connect. 2023;12(7):e220504. 37052176 10.1530/EC-22-0504 PMC10305501

[50] Allen DB . Diagnosis of growth hormone deficiency remains a judgment call—and that is good. Horm Res Paediatr. 2021;94(11-12):406‐409. 34937037 10.1159/000521628

[51] Brettell E, Högler W, Woolley R, et al The Growth Hormone Deficiency (GHD) reversal trial: effect on final height of discontinuation versus continuation of growth hormone treatment in pubertal children with isolated GHD—a non-inferiority Randomised Controlled Trial (RCT). Trials. 2023;24(1):548. 37605233 10.1186/s13063-023-07562-z PMC10440873

[52] Kamoun C, Miller VA, Feudtner C, Friedrich EA, Grimberg A. Views on short stature of female vs male endocrine pediatric patients undergoing provocative growth hormone testing and their parents. Endocr Pract. 2023;29(7):517‐524. 37088146 10.1016/j.eprac.2023.04.004 PMC10330208

